# Adjuvant Tegafur-Uracil Improves Survival in Low-Risk, Mismatch Repair Proficient Stage IIA Colon Cancer: A Propensity Score-Matched Analysis

**DOI:** 10.3390/life15121930

**Published:** 2025-12-17

**Authors:** Min-Chi Cheng, Hsu-Lin Lee, Shiue-Wei Lai, Jia-Hong Chen, Po-Huang Chen

**Affiliations:** 1 Division of Hematology and Oncology, Department of Internal Medicine, Tri-Service General Hospital, National Defense Medical University, Taipei 11490, Taiwan; chengminchi123@gmail.com (M.-C.C.); gr1027@livemail.tw (H.-L.L.); xsurfer@mail.ndmctsgh.edu.tw (S.-W.L.); 2 Department of Oncology, Tri-Service General Hospital, National Defense Medical University, Taipei 11490, Taiwan; 3 Division of Hematology and Oncology, Department of Internal Medicine, Taichung Armed Forces General Hospital, Taichung 404313, Taiwan

**Keywords:** colon cancer, tegafur-uracil (UFT), adjuvant chemotherapy, mismatch repair proficient (pMMR), propensity score matching

## Abstract

**Background**: The benefit of adjuvant chemotherapy for low-risk, mismatch repair proficient (pMMR) stage IIA colon cancer is uncertain. Surveillance is standard, but some patients relapse. Tegafur-uracil (UFT) is a low-toxicity oral option that may offer benefit; **Methods**: This retrospective study included patients with resected low-risk, pMMR stage IIA colon cancer (2013–2022). Patients receiving ≥5 postoperative UFT prescriptions were compared with those under surveillance. Propensity score matching (1:1) was applied, and disease-free survival (DFS) and overall survival (OS) were analyzed using Kaplan–Meier and Cox models with sensitivity analyses.; **Results**: Among 279 eligible patients, 71 matched pairs were analyzed. UFT reduced the risk of recurrence or death by 57% (DFS HR = 0.43, 95% CI 0.25–0.75, *p* = 0.002) and mortality by 62% (OS HR = 0.38, 95% CI 0.21–0.68, *p* < 0.001); **Conclusions**: UFT improved DFS and OS in low-risk pMMR stage IIA colon cancer, suggesting surveillance alone may undertreat some patients. Prospective trials are warranted.

## 1. Introduction

The management of Stage II colon cancer following curative resection remains a significant clinical challenge, particularly regarding the role of adjuvant chemotherapy [1]. While its benefit is well established for Stage III disease, its application in Stage II is controversial, typically reserved for patients with high-risk features such as T4 tumors, perineural invasion, or inadequate lymph node dissection [2]. Consequently, for patients with low-risk stage IIA mismatch repair proficient (pMMR) or microsatellite-stable (MSS) tumors, active surveillance is the current standard of care [3,4].

However, this observation-only approach may not be optimal for all patients. A subset of individuals with low-risk Stage IIA disease still experiences recurrence, leading to suboptimal long-term outcomes. A critical issue highlighting this treatment gap is the “survival paradox” observed in large cancer registries [5]. A Surveillance, Epidemiology, and End Results (SEER) database analysis demonstrated that patients with Stage IIA colon cancer had worse overall and cancer-specific survival compared with patients with Stage IIIA disease, who routinely receive adjuvant chemotherapy [6]. This finding suggests that a prognostically significant subgroup of Stage IIA patients is currently undertreated.

Given that these patients have undergone complete tumor resection and are considered clinically low-risk, the ideal adjuvant therapy should be effective, convenient, and associated with low toxicity [7]. Oral fluoropyrimidines, such as tegafur-uracil (UFT), represent an attractive therapeutic option [8]. UFT has a well-documented safety profile and the convenience of oral administration, making it a suitable candidate for maintenance therapy in a population where the risk-benefit ratio of intensive intravenous chemotherapy is questionable [9].

Despite this compelling rationale, the efficacy of UFT maintenance therapy in the specific subgroup of low-risk, pMMR/MSS, Stage IIA colon cancer remains poorly defined. Previous large-scale trials of UFT in broader Stage II populations have yielded inconsistent results, often failing to demonstrate a statistically significant survival benefit and rarely reporting outcomes specifically for this low-risk, MSS subgroup [10]. This knowledge gap underscores the urgent need to identify an effective, well-tolerated adjuvant strategy for these patients. Therefore, this study aimed to evaluate the real-world effectiveness of UFT maintenance therapy in patients with low-risk, pMMR/MSS Stage IIA colon cancer using a propensity score-matched cohort design.

## 2. Materials and Methods

### 2.1. Study Design and Population

This retrospective cohort study was conducted using data from the Tri-Service General Hospital (TSGH) from 2013 to 2022. TSGH is a tertiary medical center participating in the nationwide Taiwan Cancer Registry (TCR) system. The TCR is a population-based cancer registry established in 1979 and maintained by the Ministry of Health and Welfare. After the Cancer Control Act was promulgated in 2003, hospitals with 50 or more beds are required to report comprehensive cancer data, including demographic, clinical, pathological, staging, and treatment information to the central registry. The TCR has achieved data completeness exceeding 97% and high accuracy rates validated through periodic chart review audits [11,12]. The hospital-level data used in this study adhere to the standardized definitions, coding procedures, and quality assurance protocols established by the TCR [13]. Furthermore, Tri-Service General Hospital has previously conducted cancer-related research using data derived from the Taiwan Cancer Registry, demonstrating our institution’s experience with and adherence to registry-based oncology research practices [14]. The study population comprised patients with resected, low-risk, stage IIA, mismatch repair proficient (pMMR) colon cancer. High-risk features leading to exclusion were defined as T4 disease, bowel obstruction, poorly differentiated or undifferentiated histology, and the presence of lymphovascular or perineural invasion. Additional exclusion criteria included deficient mismatch repair (dMMR) status, as determined by immunohistochemistry (IHC), or microsatellite instability-high (MSI-H) status, incomplete clinical data, or loss to follow-up.

### 2.2. Cohort Definition and Covariates

Eligible patients were categorized into two cohorts based on adjuvant treatment status within 180 days of surgery. All patients meeting the eligibility criteria during the study period were included in the analysis without additional sampling. The treatment group was defined as patients who received at least five prescriptions of tegafur-uracil (UFT). The control group consisted of patients who did not receive any adjuvant chemotherapy and underwent active surveillance. For the purposes of this study, this cohort is referred to as the ‘control group’.

Baseline demographic, clinical, and pathological covariates were extracted for all patients, including gender, age, body mass index (BMI), the Adjusted Charlson Comorbidity Index (ACCI) [15], which was calculated based on International Classification of Diseases, Ninth Revision (ICD-9-CM, for cases before 2016) and Tenth Revision (ICD-10-CM, for cases from 2016 onward) codes, with the primary malignancy excluded. Other extracted covariates included Eastern Cooperative Oncology Group Performance Status (ECOG PS), tumor side, histology, differentiation grade, tumor size, carcinoembryonic antigen (CEA) level, and RAS/BRAF mutation status.

### 2.3. Statistical Analysis

All statistical analyses were conducted using R Statistical Software (version 4.2.0; R Core Team, 2022). A two-sided *p* < 0.05 was considered statistically significant. Continuous variables were assessed for normality using the Shapiro-Wilk test. Variables following normal distribution were summarized as means ±standard deviations (SD) and compared using independent *t*-tests. Variables with non-normal distributions were summarized as medians with interquartile ranges (IQR) and compared using the Mann-Whitney U test. Categorical variables were summarized as frequencies and percentages and compared using Pearson’s chi-square test or Fisher’s exact test when expected cell counts were below 5.

### 2.4. Propensity Score Matching

To address selection bias and confounding, we performed propensity score matching (PSM) to balance baseline covariates between the UFT and control groups [16]. A logistic regression model was first used to generate a propensity score for each patient, representing the probability of receiving UFT based on gender, age, ACCI, and ECOG PS. We then implemented a 1:1 nearest-neighbor matching algorithm without replacement, using a caliper width of 0.2 of the standard deviation of the logit of the propensity score. Covariate balance before and after matching was assessed using the standardized mean difference (SMD), where a value < 0.1 indicates a negligible imbalance. A Love plot was used for graphical diagnosis of balance.

As this was a retrospective study, all eligible patients meeting inclusion criteria during the study period were enrolled, and a formal a priori sample size calculation was not performed. A post hoc power analysis was conducted using G*Power software (version 3.1.9.7). With 71 matched pairs, an observed hazard ratio of 0.38 for OS, a two-sided alpha of 0.05, and an event rate of approximately 40% in the control group over the follow-up period, the study achieved a statistical power exceeding 80% to detect the observed treatment effect.

### 2.5. Outcome Analyses

The primary endpoints were disease-free survival (DFS) and overall survival (OS). Disease-free survival (DFS) was defined as the time from the date of curative surgery to the first documented event of local recurrence, distant metastasis, second primary colorectal cancer, or death from any cause, whichever occurred first. Patients without any event were censored at the date of last known follow-up. Overall survival (OS) was defined as the time from the date of surgery to death from any cause. Patients alive at the end of the study period were censored at the date of last contact.

The administrative censoring date was 1 September 2024. Patients without events were censored at the date of last documented follow-up or the administrative censoring date, whichever occurred first.

In the matched cohort, survival distributions were estimated using the Kaplan-Meier method, and differences were compared using the log-rank test. Hazard ratios (HRs) and their corresponding 95% confidence intervals (CIs) were derived from Cox proportional hazards models.

### 2.6. Sensitivity Analyses

To assess the robustness of the primary findings, several sensitivity analyses were conducted. First, a univariate Cox regression was performed in the pre-matched cohort to identify significant prognostic factors. Second, we conducted an E-value analysis to quantify the required magnitude of an unmeasured confounder to nullify the observed treatment effect [17]. Guided by these results, a final multivariable Cox regression model was constructed in the matched cohort to adjust for potential residual confounding from prognostically significant variables, including age, ECOG PS, ACCI, and histology.

### 2.7. Subgroup Analyses

To assess the consistency of treatment effect across clinically relevant patient subgroups, exploratory subgroup analyses were performed for both DFS and OS. Patients were stratified by age group (below 70 vs. above 70 years), ECOG performance status (0 vs. 1–3), enrollment period (2013–2017 vs. 2018–2022), and tumor sidedness (left vs. right). Hazard ratios with 95% confidence intervals were calculated within each subgroup using Cox proportional hazards models. Heterogeneity of treatment effect across subgroups was assessed by including an interaction term between treatment and subgroup variable in the Cox model, with a *p*-value < 0.10 considered indicative of potential heterogeneity. Given the modest sample size and multiple comparisons, these analyses were considered exploratory and hypothesis generating.

## 3. Results

### 3.1. Patient Characteristics and Propensity Score Matching

**Figure 1:**
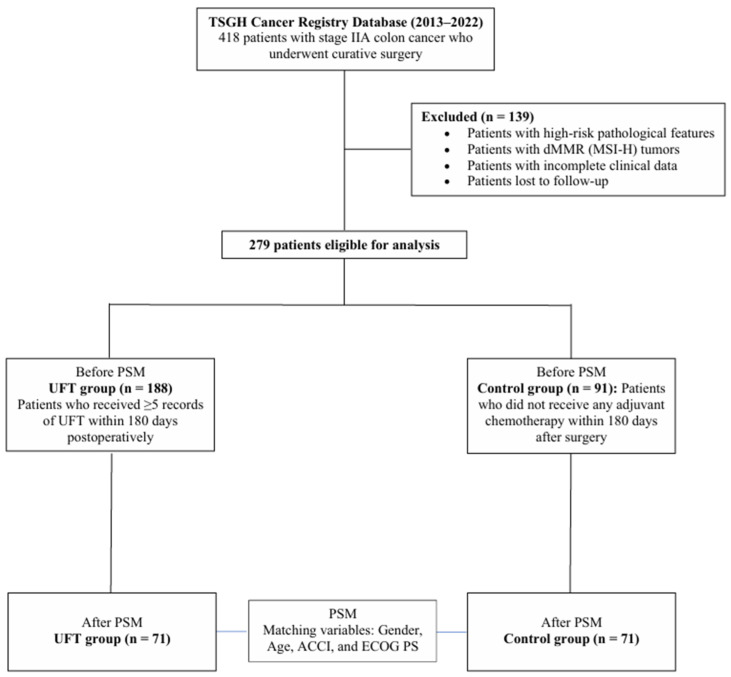
Patient disposition flowchart. Among 418 Stage IIA colon cancer patients initially identified, 279 were eligible after exclusions. Propensity score matching based on gender, age, ACCI, and ECOG PS yielded 71 matched pairs for comparison between the UFT and control groups.

From an initial cohort of 418 patients with stage IIA colon cancer identified from the TSGH Cancer Registry, 279 were deemed eligible for analysis after applying the exclusion criteria (Figure 1). This initial cohort comprised 188 patients in the UFT group and 91 in the control group.

Prior to matching, several baseline characteristics differed significantly between the two groups (Table 1). Patients in the UFT group were younger (mean age: 67.0 vs. 76.3 years, *p* < 0.001), had a lower comorbidity burden (mean ACCI: 3.96 vs. 6.07, *p* < 0.001), and had better performance status (ECOG PS, *p* < 0.001) compared to the control group.

Following 1:1 propensity score matching, 71 well-balanced matched pairs were obtained. In this matched cohort, all baseline covariates were adequately balanced, and previously noted disparities in age (*p* = 0.523), ACCI (*p* = 0.570), and ECOG PS (*p* = 0.513) were no longer statistically significant (Table 2). The successful balancing of covariates was further confirmed by the reduction in standardized mean differences for all variables, as graphically depicted in the Love plot (Supplementary Figure S1).

In the matched cohort, the median follow-up time was 53.0 months (IQR: 32.3–85.8 months).

### 3.2. Survival Outcomes in the Matched Cohort

**Figure 2:**
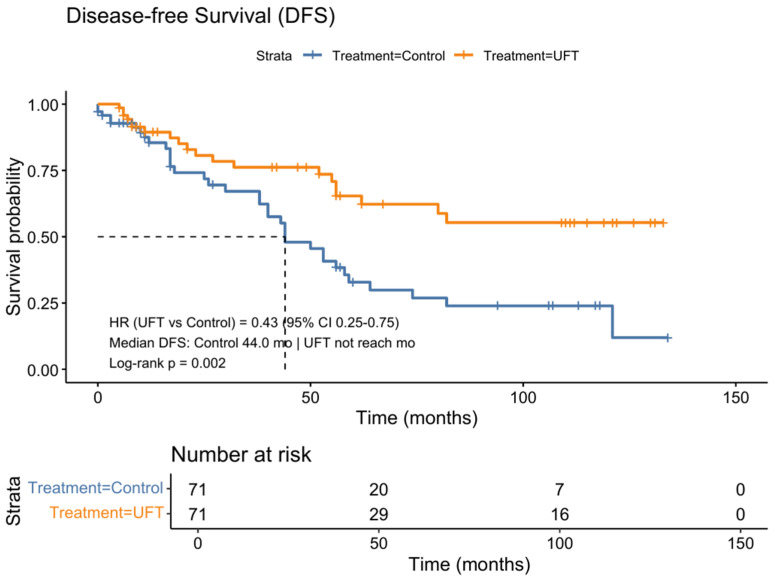
Kaplan-Meier analysis of DFS in the propensity score-matched cohort. Patients who received UFT had significantly improved DFS compared with the control group after propensity score matching. The UFT group showed a 57% reduction in the risk of recurrence or death (HR = 0.43, 95% CI 0.25–0.75), with median DFS not reached, whereas the control group had a median DFS of 44.0 months (log-rank *p* = 0.002). The dashed lines indicate the median disease-free survival.

In the matched cohort, maintenance therapy with UFT was associated with a significant improvement in survival outcomes. Kaplan-Meier analysis revealed that the UFT group had a 57% reduction in the risk of disease recurrence or death, with a median DFS that was not reached, compared to 44.0 months in the control group (HR = 0.43, 95% CI: 0.25–0.75, *p* = 0.002) (Figure 2).

**Figure 3:**
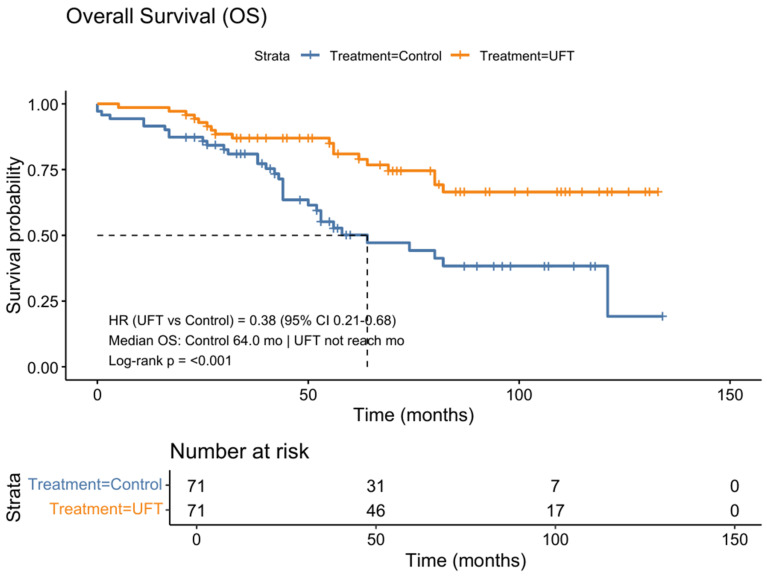
Kaplan-Meier analysis of OS in the propensity score-matched cohort. UFT was associated with significantly improved OS compared with observation. In the matched cohort, UFT reduced all-cause mortality risk by 62% (HR = 0.38; 95% CI 0.21–0.68), with median OS not reached in the UFT group versus 64.0 months in the control group (log-rank *p* < 0.001). The dashed lines indicate the median overall survival.

Similarly, UFT treatment conferred a 62% reduction in the risk of all-cause mortality. The median OS was not reached in the UFT group, versus 64.0 months in the control group (HR = 0.38, 95% CI: 0.21–0.68, *p* < 0.001) (Figure 3).

### 3.3. Prognostic Factors and Sensitivity Analyses

A univariate Cox regression on the entire pre-matched cohort identified several significant prognostic factors for survival besides UFT maintenance (Table 3). Factors associated with worse OS included advanced age (HR 1.07, *p* < 0.001), higher ACCI (HR 1.21, *p* < 0.001), poorer ECOG PS (e.g., PS 3 vs. 0, HR 6.81, *p* < 0.001), and mucinous histology (HR 3.28, *p* = 0.012).

To assess the robustness of our findings against unmeasured confounding, an E-value analysis was performed. For OS, the E-value for the HR point estimate was 4.70, with a lower limit of 2.30 for the confidence interval (Supplementary Table S1). This indicates that an unmeasured confounder would need to be associated with both UFT treatment and mortality by a risk ratio of at least 2.30 to fully attenuate the observed treatment effect. Notably, the observed HRs for severe ECOG PS (3 vs. 0) and mucinous histology exceeded this conservative E-value threshold, identifying them as powerful measured confounders.

Therefore, to confirm the robustness of our results, a final multivariable sensitivity analysis was conducted, specifically adjusting for these powerful prognostic factors. Even after adjustment for age, ACCI, ECOG PS, and histology, the protective effect of UFT remained statistically significant for both DFS (Adjusted HR = 0.44, 95% CI: 0.25–0.78, *p* = 0.005) and OS (Adjusted HR = 0.37, 95% CI: 0.20–0.66, *p* = 0.001) (Supplementary Table S2). The consistency between the crude and adjusted HRs further confirms that the observed benefit of UFT is robust and independent of these key measured confounders.

### 3.4. Subgroup-Specific Outcomes

Exploratory subgroup analyses were conducted to evaluate the consistency of UFT benefit across predefined patient subgroups (Supplementary Figures S2 and S3).

For overall survival, the protective effect of UFT was generally consistent across most subgroups. No significant heterogeneity was observed for age group (*p*-interaction = 0.994), enrollment period (*p*-interaction = 0.757), or tumor sidedness (*p*-interaction = 0.351). The treatment effect was similar between the 2013–2017 period (HR = 0.41, 95% CI: 0.20–0.86) and the 2018–2022 period (HR = 0.37, 95% CI: 0.14–0.96), suggesting that secular trends in clinical practice did not substantially influence the results. Notably, a significant interaction was observed for ECOG performance status (*p*-interaction = 0.039), with patients having ECOG PS 0 demonstrating a more pronounced survival benefit (HR = 0.07, 95% CI: 0.01–0.55) compared to those with ECOG PS 1–3 (HR = 0.54, 95% CI: 0.28–1.03).

Similar patterns were observed for disease-free survival, with consistent treatment effects across enrollment periods (*p*-interaction = 0.987) and tumor sidedness (*p*-interaction = 0.948). The interaction for ECOG performance status remained significant (*p*-interaction = 0.039), and there was a trend toward interaction for age group (*p*-interaction = 0.071).

Given the limited number of events in some subgroups and the exploratory nature of these analyses, these findings should be interpreted with caution.

## 4. Discussion

In this retrospective, propensity score-matched cohort study, we investigated the effectiveness of adjuvant tegafur-uracil (UFT) maintenance therapy in a well-defined cohort of patients with low-risk, mismatch repair proficient (pMMR), Stage IIA colon cancer. Our analysis found a significant association between UFT and improved disease-free survival (HR 0.43) and overall survival (HR 0.38) when compared to active surveillance. This association was consistent across several sensitivity analyses, including a multivariable Cox regression and an E-value analysis, the latter of which indicated a notable threshold against unmeasured confounding. These findings align with contemporary estimates of recurrence risk in stage II colon cancer, in which approximately 10–20% of patients experience disease recurrence despite being classified as clinically low risk [18].

Our results stand in contrast to those of the landmark SACURA trial, a large-scale randomized controlled study that did not find a significant DFS benefit for UFT in a broad population of Stage II colon cancer patients (HR 0.91; 95% CI 0.75–1.10) [19]. However, a critical limitation of the SACURA trial and other similar studies is the lack of specific reporting for the low-risk, pMMR/MSS subgroup. By including a heterogeneous population, any potential benefit of UFT in a precisely defined subgroup may have been diluted [20]. This interpretation aligns with prior reviews emphasizing the heterogeneity of Stage II disease and the limited absolute survival benefit associated with adjuvant chemotherapy in unselected Stage II populations [21]. By contrast, our study directly addresses this knowledge gap by focusing exclusively on the low-risk, pMMR/MSS Stage IIA subgroup, for whom the benefit of adjuvant therapy remains most uncertain. The positive outcomes of our study may be attributable to this highly selective patient cohort. Unlike broader Stage II populations, which include patients with dMMR/MSI-H tumors who are known to have a favorable prognosis and derive little or no benefit from fluoropyrimidine-based adjuvant chemotherapy [22], our study isolated a group that may be more responsive to treatment. Our findings align with a population-based cohort study from Taiwan by Chen et al., which reported a DFS benefit for UFT in Stage IIA patients (HR 0.652). However, that study did not stratify by risk or MSS status [10]. Our research builds upon their findings, suggesting that the benefit observed by Chen et al. may be primarily driven by the low-risk, pMMR/MSS subgroup that we have identified.

The clinical implications of our findings are significant. We have identified a subgroup of Stage IIA colon cancer patients who may be undertreated under current guideline recommendations but who may derive substantial benefit from a well-tolerated, orally administered maintenance therapy. The use of UFT offers a compelling risk-benefit profile for this population, avoiding the toxicities associated with more intensive intravenous chemotherapy regimens [23]. Recent studies of oral fluoropyrimidine monotherapy—including UFT/LV—have similarly demonstrated favorable survival outcomes in selected Stage II populations, further supporting the potential role of oral agents in this setting [24,25,26,27]. The strengths of our study lie in its rigorous methodology. Although retrospective in nature, the use of propensity score matching successfully balanced key baseline covariates, simulating the conditions of a randomized trial. Furthermore, the E-value analysis provided quantitative evidence that our results are robust against potential unmeasured confounders [28]. The clinical uncertainty surrounding adjuvant therapy in stage II disease has been further highlighted in recent expert commentaries, including an ESMO Open editorial emphasizing the persistent clinical dilemma in managing high-risk Stage II disease and the challenges of determining which patients may benefit from adjuvant therapy [29].

The subgroup analyses provide additional insights into the consistency and potential heterogeneity of UFT benefit. The similar treatment effects observed across the two enrollment periods (2013–2017 vs. 2018–2022) suggest that temporal confounding from evolving clinical practices did not substantially bias our findings. Interestingly, a significant interaction was observed for ECOG performance status, with patients having better baseline performance status (ECOG PS 0) deriving greater benefit from UFT maintenance therapy. This finding aligns with the biological rationale that patients with better functional reserve may be more likely to tolerate and benefit from prolonged maintenance therapy. However, given the exploratory nature of this analysis and the limited sample size, this observation requires validation in larger cohorts.

Nevertheless, this study has several important limitations that warrant consideration. First, its retrospective design is inherently prone to selection bias and residual confounding that may not be fully eliminated despite the use of PSM. Second, because this was a single-center study conducted at a tertiary referral hospital in Taiwan, the generalizability of these findings to other populations and healthcare systems may be limited. Third, although the 71 matched pairs provided adequate power for the primary analyses, the modest sample size may restrict the detection of smaller effect sizes or differences within specific subgroups. Fourth, the long study period (2013–2022) raises the possibility of temporal confounding related to evolving surgical practices, surveillance protocols, and supportive care standards, which could not be fully accounted for. Fifth, detailed information on treatment adherence, cumulative UFT dose, adverse events, and patient-reported outcomes was not systematically collected, limiting our ability to comprehensively evaluate the tolerability and overall risk-benefit profile of UFT. Lastly, due to sample size constraints, we were unable to perform subgroup analyses that might help identify which patients are most likely to benefit from UFT maintenance therapy.

It is important to interpret these findings within the context of current clinical practice guidelines. Both the American Society of Clinical Oncology (ASCO) and European Society for Medical Oncology (ESMO) guidelines recommend observation without adjuvant chemotherapy for patients with low-risk, pMMR stage II colon cancer [2,30], based on the lack of definitive evidence from randomized trials in this specific population. Our findings do not contradict these recommendations but rather generate a hypothesis that warrants prospective validation. The observed magnitude of benefit (HR 0.38 for OS) is notably larger than typically seen in adjuvant therapy trials, which may reflect residual confounding inherent to observational studies despite our methodological efforts to minimize bias. Therefore, while our results suggest that a subset of low-risk stage IIA patients may benefit from UFT maintenance, this hypothesis requires confirmation in prospective, randomized trials before any modification to standard practice guidelines can be recommended.

## 5. Conclusions

In conclusion, the findings from our retrospective study suggest a potential association between adjuvant UFT maintenance therapy and improved survival outcomes in a select population of patients with low-risk, pMMR/MSS, Stage IIA colon cancer. While limited by its retrospective design and modest sample size, this hypothesis-generating result suggests that an observation-only approach may not be optimal for all patients in this subgroup. Therefore, these preliminary findings highlight the need for further investigation. Prospective, randomized trials are warranted to definitively establish the role and benefit of UFT in this specific clinical setting.

## Figures and Tables

**Table 1:** Baseline characteristics of patients before propensity score matching.

Variable	Level	Control (*n* = 91)	UFT (*n* = 188)	*p*-Value	SMD
Gender	Female/Male	44 (48.4%)/47 (51.6%)	91 (48.4%)/97 (51.6%)	0.993	0.001
Age (years)		76.27 ± 12.31	66.99 ± 11.76	<0.001	−0.771
BMI (kg/m^2^)		24.00 ± 3.59 Median (IQR) † 23.44 (22.67–24.59)	23.64 ± 2.86 Median (IQR) † 23.44 (22.34–24.25)	0.734	0.050
ACCI (Adjusted Charlson Comorbidity Index)		6.07 ± 3.73	3.96 ± 2.55	<0.001	−0.660
ECOG PS (0–4)	0/1/2/3/4	33 (36.3%)/37 (40.7%)/13 (14.3%)/6 (6.6%)/2 (2.2%)	112 (59.6%)/57 (30.3%)/17 (9.0%)/2 (1.1%)/0 (0.0%)	<0.001 #	0.687
Tumor side	Left/Right	32 (35.2%)/59 (64.8%)	93 (49.5%)/95 (50.5%)	0.024	0.407
Histology	Adenocarcinoma/Mucinous adenocarcinoma	86 (94.5%)/5 (5.5%)	184 (97.9%)/4 (2.1%)	0.157 #	0.269
Differentiation	Moderately/Well differentiated	85 (93.4%)/6 (6.6%)	168 (89.4%)/20 (10.6%)	0.276	0.197
Tumor size (mm)		61.35 ± 101.52 Median (IQR) † 48.50 (35.25–59.75)	61.27 ± 121.19 Median (IQR) † 42.00 (32.00–55.00)	0.995	−0.001
CEA (ng/mL)		8.12 ± 13.71	8.21 ± 17.69	0.970	0.006
RAS mutation	Wild-type/Mutant	58 (65.2%)/31 (34.8%)	125 (66.5%)/63 (33.5%)	0.828	0.039
BRAF mutation	Wild-type/Mutant	91 (100.0%)/0 (0.0%)	184 (97.9%)/4 (2.1%)	—	0.253
UFT dose (mg/day) [UFT group only]	100/200/300/400/600		3 (1.6%)/18 (9.6%)/40 (21.3%)/123 (65.4%)/4 (2.1%)		
UFT maintenance time (months) [UFT group only]			17.25 ± 8.28		

Abbreviations: UFT, tegafur-uracil; ACCI, Adjusted Charlson Comorbidity Index; ECOG PS, Eastern Cooperative Oncology Group Performance Status; CEA, carcinoembryonic antigen; RAS, rat sarcoma viral oncogene homolog; BRAF, v-raf murine sarcoma viral oncogene homolog B. Continuous variables are presented as the mean ± standard deviation; categorical variables are presented as n (%). Continuous variables were compared using the independent *t*-test or Mann-Whitney U test; categorical variables were compared using the chi-square test or Fisher’s exact test. # *p*-values were calculated using Fisher’s exact test or Kruskal-Wallis test as appropriate. † Median (interquartile range) reported for non-normally distributed variables; Mann-Whitney U test was used for comparison. — *p*-value not computed due to zero counts in one group.

**Table 2:** Baseline characteristics of patients after propensity score matching.

Variable	Level	Control (*n* = 71)	UFT (*n* = 71)	*p*-Value	SMD
Gender	Female/Male	34 (47.9%)/37 (52.1%)	36 (50.7%)/35 (49.3%)	0.737	0.080
Age (years)		73.87 ± 12.79	72.41 ± 14.38	0.523	−0.107
BMI (kg/m^2^)		24.20 ± 3.79 Median (IQR) † 23.40 (23.40–24.75)	23.38 ± 2.86 Median (IQR) † 23.40 (22.70–24.05)	0.572	0.095
ACCI (Adjusted Charlson Comorbidity Index)		5.30 ± 3.38	4.99 ± 3.10	0.570	−0.096
ECOG PS (0–3)	0/1/2/3	32 (45.1%)/25 (35.2%)/9 (12.7%)/5 (7.0%)	28 (39.4%)/32 (45.1%)/9 (12.7%)/2 (2.8%)	0.513 #	0.303
Tumor side	Left/Right	22 (31.0%)/49 (69.0%)	30 (42.3%)/41 (57.7%)	0.163	0.331
Histology	Adenocarcinoma/Mucinous adenocarcinoma	67 (94.4%)/4 (5.6%)	68 (95.8%)/3 (4.2%)	1.000 #	0.092
Differentiation	Moderately/Well differentiated	65 (91.5%)/6 (8.5%)	59 (83.1%)/12 (16.9%)	0.130	0.359
Tumor size (mm)		61.75 ± 114.60 Median (IQR) † 45.00 (35.00–55.75)	58.39 ± 113.83 Median (IQR) † 44.00 (35.00–52.50)	0.861	−0.029
CEA (ng/mL)		8.56 ± 14.18	10.41 ± 18.35	0.525	0.113
RAS mutation	Wild-type/Mutant	47 (68.1%)/22 (31.9%)	43 (60.6%)/28 (39.4%)	0.351	0.223
BRAF mutation	Wild-type/Mutant	71 (100.0%)/0 (0.0%)	70 (98.6%)/1 (1.4%)	—	0.238
UFT dose (mg/day) [UFT group only]	200/300/400/600	NA	8 (11.3%)/15 (21.1%)/47 (66.2%)/1 (1.4%)		
UFT maintenance time (months) [UFT group only]		NA	15.72 ± 8.65		

Abbreviations: UFT, tegafur-uracil; ACCI, Adjusted Charlson Comorbidity Index; ECOG PS, Eastern Cooperative Oncology Group Performance Status; CEA, carcinoembryonic antigen; RAS, rat sarcoma viral oncogene homolog; BRAF, v-raf murine sarcoma viral oncogene homolog B. Continuous variables are presented as the mean ± standard deviation; categorical variables are presented as n (%). Continuous variables were compared using the independent *t*-test or Mann-Whitney U test; categorical variables were compared using the chi-square test or Fisher’s exact test. # *p*-values were calculated using Fisher’s exact test or Kruskal-Wallis test as appropriate. † Median (interquartile range) reported for non-normally distributed variables; Mann-Whitney U test was used for comparison. — *p*-value not computed due to zero counts in one group.

**Table 3:** Univariate Cox regression analysis of factors associated with DFS and OS.

Variable	DFS HR (95% CI)	*p*-Value	OS HR (95% CI)	*p*-Value
UFT maintenance	0.43 (0.25–0.75)	0.003	0.38 (0.21–0.68)	0.001
Age (years)	1.05 (1.03–1.08)	<0.001	1.07 (1.04–1.10)	<0.001
Gender	1.32 (0.78–2.25)	0.304	1.20 (0.69–2.08)	0.521
BMI (kg/m^2^)	0.99 (0.93–1.05)	0.74	0.99 (0.92–1.06)	0.668
ACCI	1.20 (1.12–1.28)	<0.001	1.21 (1.12–1.30)	<0.001
ECOG PS				
ECOG PS (1 vs. 0)	1.78 (0.91–3.47)	0.09	1.89 (0.96–3.74)	0.066
ECOG PS (2 vs. 0)	3.55 (1.58–7.99)	0.002	2.71 (1.19–6.21)	0.018
ECOG PS (3 vs. 0)	6.30 (2.20–18.03)	0.001	6.81 (2.36–19.65)	<0.001
Tumor side (Right vs. Left)	0.88 (0.51–1.51)	0.64	0.76 (0.43–1.33)	0.333
Histology (Mucinous vs. Adenocarcinoma)	2.59 (1.03–6.52)	0.044	3.28 (1.29–8.32)	0.012
Differentiation (Well vs. Moderate differentiated)	0.47 (0.19–1.18)	0.109	0.44 (0.16–1.22)	0.113
Tumor size (mm)	1.00 (1.00–1.00)	0.056	1.00 (1.00–1.00)	0.107
CEA (ng/mL)	1.01 (0.99–1.02)	0.239	1.01 (0.99–1.02)	0.214
RAS mutation	1.28 (0.73–2.23)	0.394	1.30 (0.73–2.32)	0.371

Abbreviations: DFS, disease-free survival; OS, overall survival; UFT, tegafur–uracil; ACCI, Adjusted Charlson Comorbidity Index; ECOG PS, Eastern Cooperative Oncology Group Performance Status; CEA, carcinoembryonic antigen; RAS, rat sarcoma viral oncogene homolog.

## Data Availability

The data presented in this study are available upon request from the corresponding authors due to privacy and ethical restrictions.

## Supplementary Materials

The following supporting information can be downloaded at: https://www.mdpi.com/article/10.3390/life15121930/s1, Figure S1: Love Plot (Covariate balance after PSM using a Love plot); Figure S2: Forest plot of subgroup analyses for overall survival; Figure S3: Forest plot of subgroup analyses for disease-free survival. Table S1: E-value sensitivity analysis for unmeasured confounding; Table S2: Multivariable sensitivity analysis of UFT effect after adjusting for confounders identified by E-value analysis.

## Abbreviations

The following abbreviations are used in this manuscript:

ACCI	Adjusted Charlson Comorbidity Index
ECOG PS	Eastern Cooperative Oncology Group Performance Status
ICD	International Classification of Diseases
MSS	Microsatellite-stable
PSM	Propensity Score Matching
pMMR	Mismatch repair proficient
UFT	Tegafur-uracil
