# Supplementary material for: Adjuvant Tegafur-Uracil Improves Survival in Low-Risk, Mismatch Repair Proficient Stage IIA Colon Cancer: A Propensity Score-Matched Analysis
Source: Life (Basel). 2025 Dec 17;15(12):1930. doi: 10.3390/life15121930 (PMC12735150; doi:10.3390/life15121930)
Supplement: Supplementary file 1 [file life-15-01930-s001.zip › Supplementary Materials.pdf]

Supplementary Materials

Supplementary Figure S1. Love Plot (Covariate balance after PSM using a Love plot)

Supplementary Figure S2. Forest plot of subgroup analyses for overall survival.

Supplementary Figure S3. Forest plot of subgroup analyses for disease-free survival.

Supplementary Table S1. E-value sensitivity analysis for unmeasured confounding

Supplementary Table S2. Multivariable sensitivity analysis of UFT effect after adjusting for confounders identified by E-value analysis

Supplementary Figure S1. Love Plot (Covariate balance using a Love plot)

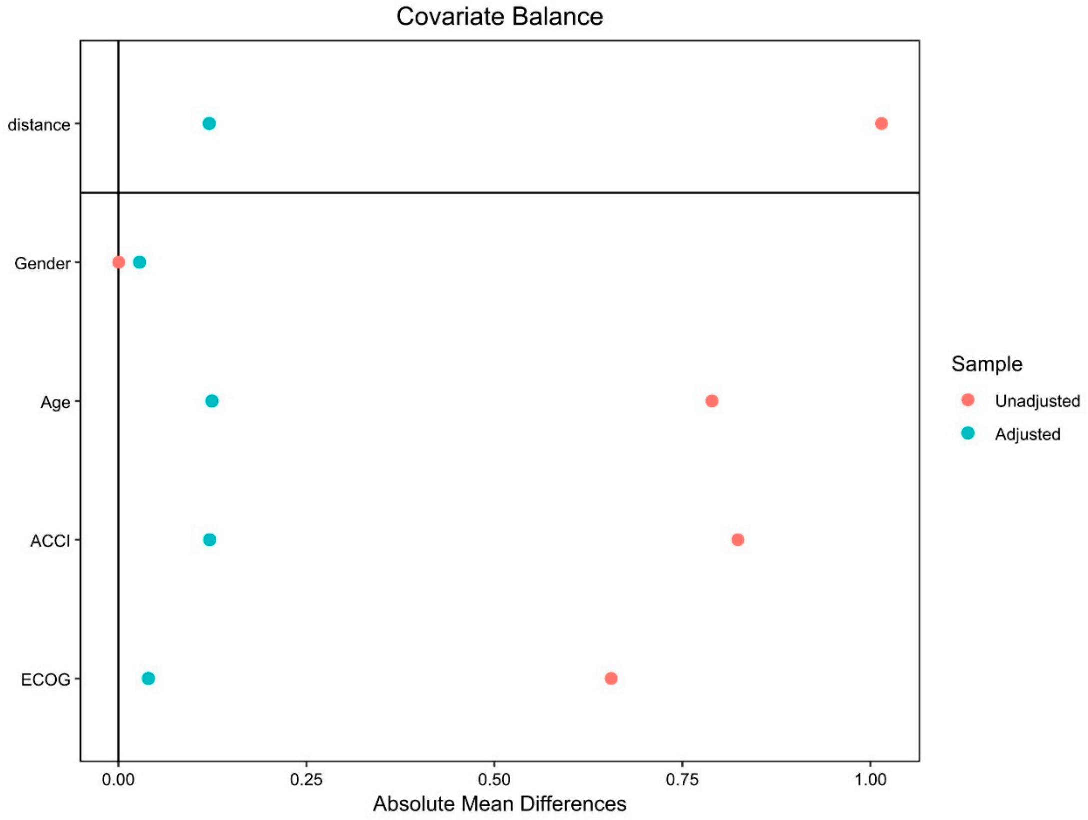

Absolute standardized mean differences are displayed for each covariate, including gender, age, ACCI, and ECOG performance status, as well as the propensity score (distance). Substantial imbalance was present in the unadjusted sample, particularly for age, ACCI, and ECOG performance status. After matching, all covariates demonstrated marked improvement in balance, with standardized mean differences reduced to below commonly accepted thresholds, indicating successful mitigation of baseline differences between treatment groups.

Supplementary Figure S2. Forest plot of subgroup analyses for overall survival.

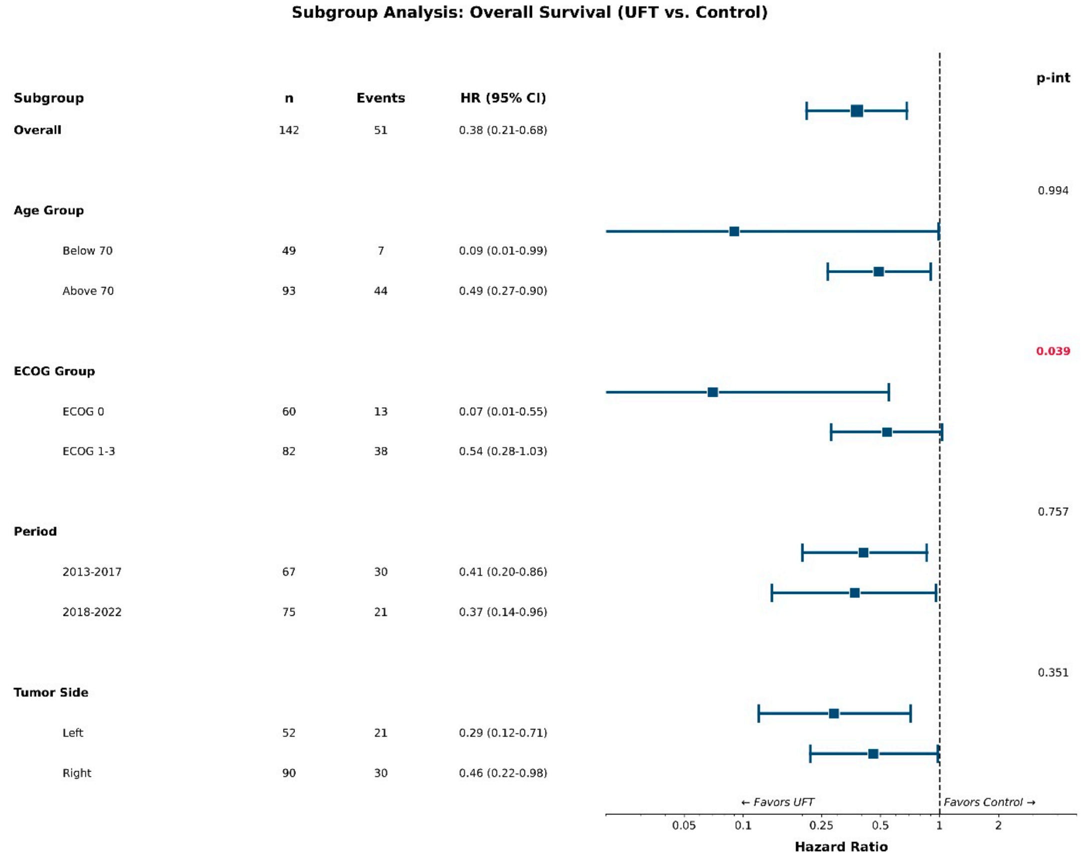

Hazard ratios with 95% confidence intervals for UFT versus control are presented for each predefined subgroup. Subgroups evaluated include age group (below 70 vs. above 70 years), ECOG performance status (0 vs. 1–3), enrollment period (2013–2017 vs. 2018–2022), and tumor sidedness (left vs. right). The dashed vertical line denotes the null effect (HR = 1.0). P-interaction values evaluate heterogeneity of treatment effects across subgroups. A significant interaction was observed for ECOG performance status (p = 0.039), indicating that patients with better baseline functional status derived a greater survival benefit from UFT maintenance therapy.

Supplementary Figure S3. Forest plot of subgroup analyses for disease-free survival.

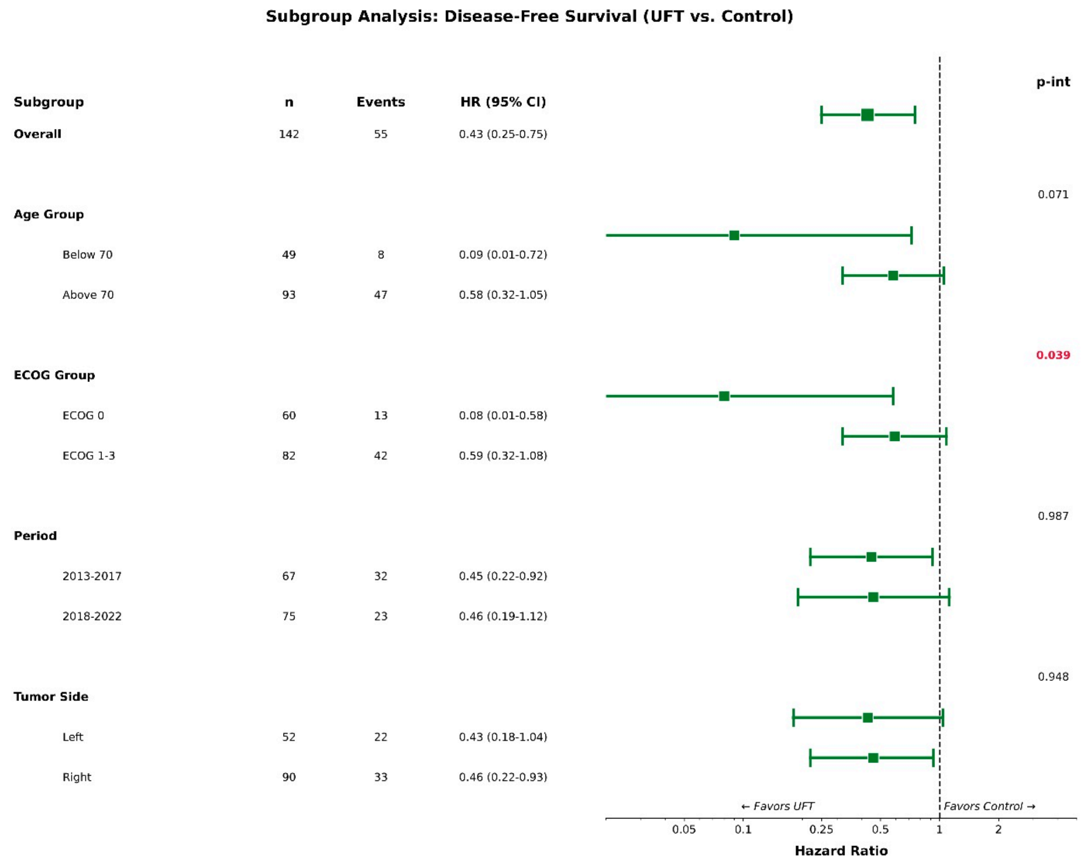

Hazard ratios with 95% confidence intervals for UFT versus control are presented for each predefined subgroup. Subgroups include age group (below 70 vs. above 70 years), ECOG performance status (0 vs. 1–3), enrollment period (2013–2017 vs. 2018–2022), and tumor sidedness (left vs. right). The dashed vertical line denotes the null effect (HR = 1.0). P-interaction values evaluate heterogeneity of treatment effects across subgroups. A significant interaction was observed for ECOG performance status (p = 0.039), and a trend toward interaction was noted for age group (p = 0.071).

Supplementary Table S1. E-value sensitivity analysis for unmeasured confounding

Part A: E-values for Treatment Effect			
Outcome	HR (95% CI)	Point Estimate E-value	Lower CI E-value
Disease-Free Survival	0.43 (0.25–0.75)	4.08	2.00
Overall Survival	0.38 (0.21–0.68)	4.70	2.30

Part B: Comparison with Measured Confounders (Overall Survival)			
Variable	Univariable HR (95% CI)	Adjusted in Multivariable Model	Exceeds E-value (3.14)?
E-value threshold for OS: 3.14 (lower CI E-value)			
ECOG PS (3 vs 0)	6.81 (2.36–19.65)	Yes	Yes*
Histology (Mucinous vs Adenocarcinoma)	3.28 (1.29–8.32)	Yes	Yes*
ECOG PS (2 vs 0)	2.71 (1.19–6.21)	Yes	No
ECOG PS (1 vs 0)	1.89 (0.96–3.74)	Yes	No
ACCI (per point increase)	1.21 (1.12–1.30)	Yes	No
Age (per year increase)	1.07 (1.04–1.10)	Yes	No

Abbreviations: HR, hazard ratio; CI, confidence interval; ECOG PS, Eastern Cooperative Oncology Group Performance Status; ACCI, Adjusted Charlson Comorbidity Index.

E-value interpretation: The E-value represents the minimum strength of association (on the risk ratio scale) that an unmeasured confounder would need to have with both UFT maintenance treatment and the outcome to fully explain away the observed treatment effect, conditional on measured covariates. For overall survival, an unmeasured confounder would need to be associated with both treatment assignment and mortality by a risk ratio of ≥4.70 (for the point estimate) or ≥2.30 (for the confidence interval lower bound) to negate the observed protective effect.

*Although ECOG PS 3 and mucinous histology demonstrate associations exceeding the conservative E-value threshold (lower CI E-value = 2.30), both variables were adjusted for in the multivariable Cox regression model, thereby controlling for their confounding effects.

Note: E-values were calculated using the formula: $RR + \sqrt{(RR \times (RR - 1))}$, where $RR = 1/HR$ for protective effects ($HR < 1$). The comparison uses the lower confidence interval E-value (2.30 for OS) as a conservative threshold, representing the minimum confounding strength needed even under the most pessimistic estimate of the treatment effect within the confidence interval.

Supplementary Table S2. Multivariable sensitivity analysis of UFT effect after adjusting for confounders identified by E-value analysis

Outcome	n	Crude Analysis		Adjusted Analysis*	
		HR (95% CI)	p-value	HR (95% CI)	p-value
**Disease-Free Survival**	142	**0.43 (0.25–0.75)**	**0.003**	**0.44 (0.25–0.78)**	**0.005**
**Overall Survival**	142	**0.38 (0.21–0.68)**	**0.001**	**0.37 (0.20–0.66)**	**0.001**

Abbreviations: HR, hazard ratio; CI, confidence interval; UFT, tegafur-uracil.
***Adjusted for:** Age (continuous), ECOG performance status (0, 1, 2, 3), Adjusted Charlson Comorbidity Index (ACCI, continuous), and histology (mucinous vs. adenocarcinoma).
Note: Analysis performed in propensity score-matched cohort. The minimal difference between crude and adjusted hazard ratios (change of 2–3%) indicates excellent balance of measured confounders achieved through propensity score matching. Treatment effect remained statistically significant in both univariate and multivariable models, confirming the robustness of findings.
